# Alcohol Consumption and Health Outcomes Among Men in a Rural HIV-Endemic Cohort in South Africa

**DOI:** 10.12688/wellcomeopenres.24185.2

**Published:** 2026-01-27

**Authors:** Alison C. Castle, Sheela V. Shenoi, Kobus Herbst, Emily B. Wong, Thumbi Ndung'u, Willem Hanekom, Judith A. Hahn, Mark J. Siedner, Vukuzazi Study Team

**Affiliations:** 1 Africa Health Research Institute, Durban, KwaZulu-Natal, 3935, South Africa; 2 Division of Infectious Diseases, Massachusetts General Hospital, Boston, Massachusetts, USA; 3 Harvard Medical School, Boston, Massachusetts, USA; 4 Yale University School of Medicine, New Haven, Connecticut, USA; 5 University of KwaZulu-Natal, Durban, South Africa; 6 DSI-SAMRC South African Population Research Infrastructure Network, Durban, South Africa; 7 Division of Infectious Diseases, The University of Alabama, Birmingham, Alabama, USA; 8 Division of Infection and Immunity, University College London, London, England, UK; 9 Division of HIV, ID, & Global Medicine, University of California San Francisco, San Francisco, California, USA

**Keywords:** alcohol, men, South Africa, HIV, mortality

## Abstract

**Background:**

In South Africa, men experience lower HIV care engagement and retention than women. Men who consume alcohol face greater barriers to care and may represent a population with significant unmet health needs. We investigated associations between alcohol consumption, HIV clinical outcomes, and all-cause mortality among men in rural South Africa.

**Methods:**

We analyzed data from men in a population-based health screening in KwaZulu-Natal linked to longitudinal clinical and mortality data. Our primary exposure was hazardous alcohol use, defined as an AUDIT-C score ≥4. Our primary outcomes were: living with HIV, awareness of HIV positive status, virologic suppression (<40 copies/mL), advanced HIV disease (CD4 count<200 cells/mm ^3^), healthcare access (clinic visit <12 months), and all-cause mortality. Poisson regression models were used for HIV outcomes and clinic follow-up; Cox proportional hazard models were used for mortality. Models were adjusted for clinical and demographic confounders. In sensitivity analyses, alcohol use was redefined as any use in the past 12 months.

**Results:**

Among 5,771 men (median age 36.2 years, SD 18.9), 10% met criteria for hazardous alcohol use with 28% reporting any alcohol use in the past 12 months. Hazardous alcohol use was not associated with HIV prevalence (aPR 0.96 95%CI 0.83-1.12, p=0.63), awareness of HIV diagnosis (aPR 1.25, 95%CI 0.88–1.77, p=0.22), virologic suppression (aPR 0.92, 95%CI 0.82–1.03, p=0.14), or advanced HIV disease (aPR 0.71, 95%CI 0.40–1.26, p=0.24). Clinic retention was low overall (56% hazardous alcohol vs. 63% no/low-risk alcohol, p=0.092) and similar between groups in adjusted models. Over a median 3.4 years, 7.3% of men with HIV died, with no differences by hazardous alcohol use. Results were similar when considering any alcohol use as the exposure.

**Conclusions:**

Hazardous alcohol use among rural South Africa men was not associated with HIV prevalence, HIV clinical outcomes, retention in care, or short-term mortality.

## Plain Language summary

**Alcohol use and hiv health outcomes among men in rural South Africa**

Men in South Africa are less likely than women to engage in HIV care. Men who drink alcohol may face even greater barriers to staying healthy. This study looked at how alcohol use affects HIV status, healthcare access, and survival among men in a rural area of KwaZulu-Natal.

Researchers used data from over 5,700 men who participated in a community health screening. They linked this information to long-term clinical and survival records. Hazardous alcohol use was defined as drinking patterns that could lead to health problems.

The study found that hazardous drinking was common—about 10% of men met the criteria. However, hazardous alcohol use was not linked to a higher rate of HIV infection, lower rates of HIV treatment success, worse immune system health, missing clinic visits, or early death.

Overall, fewer than two-thirds of men stayed connected to healthcare after screening, regardless of whether they drank alcohol. Mental health conditions like anxiety and depression, and being employed, were more strongly linked to poor health outcomes than alcohol use.

These findings suggest that while alcohol use is common, it may not directly impact HIV outcomes in the era of modern treatment. Addressing broader issues, such as mental health support and easier access to care, may help improve health outcomes for men in this region.

## Introduction

In South Africa, men experience disproportionately lower rates of HIV care engagement compared to women ^1– 3^. Despite the free and widespread availability of antiretroviral therapy (ART) in public health clinics, men are less likely to know their HIV status, initiate treatment, and achieve viral suppression ^4^. These disparities are particularly pronounced in the northeast region of KwaZulu-Natal, where men residing in rural settlements had a twofold higher risk for living with HIV compared to men in other settlement types ^5, 6^. Contributing barriers include inconvenient clinic hours, stigma, and harmful masculinity norms that promote invulnerability, and reliance on subjective health measures such as “feeling healthy” or a partner’s HIV status, which ultimately discourage men from seeking care ^7– 10^.

Hazardous alcohol use has been identified as a key driver of these gendered health disparities in South Africa, particularly among men, where alcohol use has been linked to HIV acquisition risk, delayed care engagement, and poorer health outcomes in multiple South African studies ^11, 12^. The country has one of the highest per capita alcohol consumption rates in Africa, with patterns of binge drinking and hazardous use especially common among men ^13, 14^. The 2020 Global Burden of Disease Study reported that 47% of South African men consume alcohol, marking a 4% increase since 1990 ^15^. The historical roots of hazardous alcohol use can be traced to the apartheid-era “dop system”, which institutionalized alcohol misuse by compensating Black laborers—particularly in agricultural settings—with alcohol instead of wages ^16^. This exploitative practice established intergenerational patterns of alcohol dependency, which persist in post-apartheid South Africa ^16^. Informal drinking venues, such as shebeens, remain central to social and economic life, but are also associated with high-risk sexual behavior, greater alcohol consumption, multiple sexual partners, and lower condom use ^17– 19^.

Previous studies have documented the link between alcohol use and high-risk sexual behaviors ^20^, challenges with ART adherence ^21^, and HIV-related mortality ^22^. However, much of this evidence comes from clinic-based cohorts or cross-sectional behavioral studies conducted before the widespread use of modern integrase inhibitor–based ART regimens. Because these regimens achieve rapid viral suppression and have higher barriers to resistance, the clinical consequences of intermittent nonadherence associated with alcohol use may differ in the current ART era. As a result, the relationship between alcohol use and longitudinal HIV clinical outcomes at the population level remains incompletely understood. Leveraging longitudinal data from the Africa Health Research Institute’s Health and Demographic Surveillance System linked with clinical and alcohol use data from a large community-based health screening, this study provides a novel opportunity to examine whether and how alcohol use continues to shape HIV-related health outcomes, healthcare engagement, and all-cause mortality in this high-burden region.

In this study, we conducted a longitudinal analysis to examine the association between alcohol use and HIV serostatus, awareness of HIV seropositivity, virologic suppression, CD4 cell counts, clinical retention, and all-cause mortality. Our findings will support the development male-tailored interventions in South Africa to address both hazardous alcohol use and HIV related-outcomes in a population largely absent from the public health domain.

## Methods

### Study design and procedures

We conducted a secondary analysis of data from the Vukuzazi study, a large community-based, cross-sectional health screening program that enrolled participants between May 2018 and March 2020 in the uMkhanyakude District, KwaZulu-Natal, South Africa ^23, 24^. Briefly, all residents of the Africa Health Research Institute’s Health and Demographic Surveillance System (HDSS) aged 15 years or older were visited at home and invited to participate in a mobile health screening that traveled through the study catchment area. The HDSS, which spans 845km ^2^ and includes approximately 150,000 residents characterized through tri-annual household surveys, is comprised of 100% individuals of Black African descent, 58% of adults with unemployment, and 66% with access to piped water in their homes ^23, 25^. During the study, participants completed questionnaires on demographic measures, current medications, alcohol use behaviors, and medical history including HIV. Venipuncture whole blood samples were collected for HIV (Genscreen Ultra HIV Ag-Ab enzyme immunoassay (Bio-Rad)) and hemoglobin A1c (VARIANT II TURBO Hemoglobin test system (Bio-Rad, Marnes-la-Coquette, France)). Participants with a positive HIV immunoassay had a reflex HIV-1 RNA viral load performed (Abbott RealTime HIV-1 Viral Load, Abbott, Illinois, USA) and CD4 cell count (in-house flow cytometry).

### Primary exposure and definitions

Our primary exposure of interest was hazardous alcohol use, defined by the validated Alcohol Use Disorders Identification Test-Consumption (AUDIT-C) questionnaire ^26^. Responses to the following questions were used to map onto and calculate the AUDIT-C scores: (1) alcohol consumption in the past 12 months; (2) the number of standard drinks consumed monthly; (3) the number of drinks consumed in a single sitting; and (4) the frequency of consuming six or more drinks in a sitting in the past month. Alcohol use was categorized as no/low-risk (AUDIT-C score 0–3) or hazardous (AUDIT-C score 4–12), based on thresholds established in prior studies which established higher AUDIT-C scores to increased risks of alcohol use disorders and adverse health outcomes ^27^.

In sensitivity analyses, alcohol use was dichotomized as (1) no alcohol use or (2) current alcohol use in the past 12 months. This approach was informed by evidence that participants, particularly those living with HIV, may under-report alcohol consumption due to social desirability bias ^28^. For example, a study in Uganda found that 13% of participants who denied alcohol use tested positive for alcohol biomarkers (PEth), with under-reporting strongly associated with higher social desirability scores ^28^.

### Outcomes and definitions

We selected six clinically relevant outcomes: 1) HIV serostatus, determined using enzyme-linked immunosorbent assay (ELISA) testing and considered among the entire cohort, 2) awareness of positive HIV serostatus at the health screening considered among those who were living with HIV; 3) viral load suppression defined as an HIV-1 RNA viral load of <40 copies/mL; 4) advanced HIV disease, defined as a CD4 T-cell <200 cells/ml; 5) access to clinic care, considered among men living with HIV and defined as attendance at one of the 11 outpatient public clinics within 12 months after attending the health fair, and based on expectations a minimum of one annual visit for HIV care, and 6) all-cause mortality, considered among men living with HIV.

A central element of the Vukuzazi study was referral of participants with any new or uncontrolled disease (HIV, tuberculosis, hypertension, diabetes) to local public health clinics for establishment or re-engagement in care. Referrals were coordinated through AHRI study nurses stationed at local clinics. The HDSS centralized linkage database, updated by research data clerks in the 11 local primary healthcare clinics and referral hospital, enable monitoring of all clinic visits for HDSS residents. All-cause mortality was captured through household follow-up visits conducted tri-annually as part of the HDSS. Notably, individuals who out-migrate from the surveillance area are still accounted for if family members remaining in the area can confirm their vital status. Mortality data were linked to HDSS participant identifiers and were available up until August 2023.

### Covariates

Potential confounders of the association between alcohol use and HIV clinical outcomes, clinic retention, and mortality included age, socioeconomic status, education level, employment status, and anxiety/depression. Participants were categorized into five age groups based on life stages and distribution of the cohort: 15–24 years, 25–34 years, 35–49 years, and 50+ years. This categorization was chosen to account for potential non-linear relationships between age and HIV health outcomes. A new diagnosis (e.g., HIV, tuberculosis, hypertension, diabetes) at the health screening prompted referrals to the public health clinics, therefore this covariate was also included in our clinic retention models. Socioeconomic status was estimated using household asset ownership data collected within two years of the Vukuzazi enrollment date through the HDSS to generate a relative wealth index, as developed by Filmer and Pritchett ^29^. Employment status was dichotomized as employed or unemployed, while highest completed education level was categorized as none, primary school (grades 1–8), or high school. No male participants in the analytic cohort reported college-level attainment. Anxiety was assessed through self-report using the validated EQ-5D-3L questionnaire, with participants indicating if they were “not anxious or depressed,” “moderately anxious or depressed,” or “extremely anxious or depressed.” ^30^ For this analysis, anxiety/depression was dichotomized as present for participants reporting moderate or extreme anxiety/depression ^30^.

### Statistical analysis

For the purposes of our analyses, we included only men who were enrolled in the Vukuzazi study, had completed alcohol use questionnaires, and had valid HIV serostatus results. We first described participants’ characteristics stratified by alcohol use and explored differences between groups using Kruskal-Wallis testing. To explore the associations between alcohol use and HIV-clinical outcomes (e.g., HIV serostatus, new HIV diagnosis, viral suppression, CD4 <200 cells/mL), we fitted Poisson regression models, with and without adjustment for potential confounders. The following covariates were included: age, socioeconomic status, education level, employment status, and anxiety/depression. Smoking demonstrated collinearity with alcohol use and was not included in the final models.

We then conducted survival analyses with the Kaplan-Meier estimator to compare 1) the time to clinic visit after the health screening and 2) time to death, stratified by hazardous alcohol use. We fitted Poisson regression models for clinic follow-up within 12 months after the health screening, with and without adjustment for age, socioeconomic status, education level, anxiety/depression, and a new diagnosis at the Vukuzazi health screening. Lastly, Cox proportional hazard models were fitted to assess the relationship between hazardous alcohol use and mortality, in univariate and adjusted models. The proportional hazards assumption was assessed using Schoenfeld residuals and log-log survival plots for all variables included in the Cox proportional hazards model. All statistical analyses were performed using Stata (Version 17, StataCorp, College Station, Texas, USA), with statistical significance set at p<0.05.

## Results

### Sample characteristics

**Figure 1.**
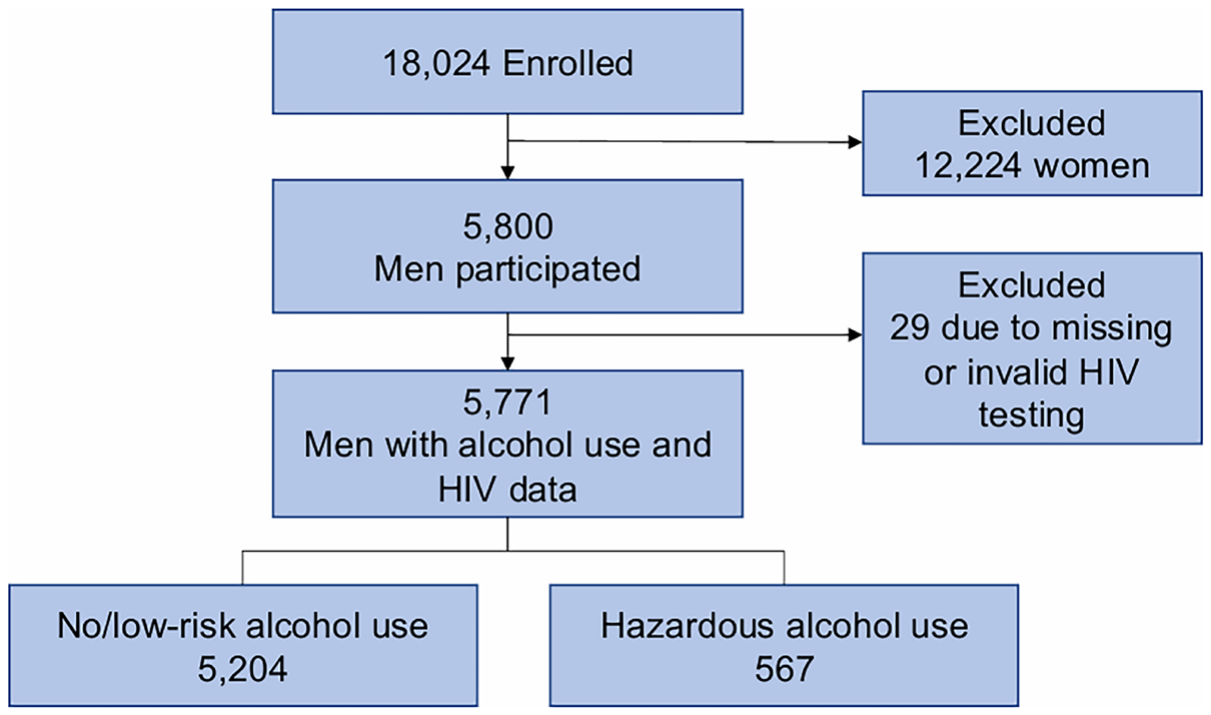
Analytic Cohort Flow Diagram. This figure outlines the inclusion process for the study cohort, from the total adult population in the Vukuzazi Study to the final analytic sample of 5,771 men who completed both alcohol use assessments and HIV testing.

Among 34,721 eligible adults in the catchment area, 18,024 (51.9%) were enrolled in the Vukuzazi study. Women (OR 2.03, 95% CI 1.94-2.12; p<0.001) and individuals aged 50 years or older (OR 2.01, 95% CI 1.92-2.11; p<0.001) were more likely to attend the Vukuzazi study compared to men and younger participants. Of the enrolled participants, 5,800 were men, of whom 5,771 (99.5%) completed both alcohol use questionnaires and HIV testing (29 excluded due to incomplete HIV testing) ( Figure 1).

The average age of male participants was 36.2 years (SD 18.9), with men reporting hazardous alcohol use being older on average compared to those with no/low risk alcohol use (40.6 versus 35.7 years, respectively) ( Table 1). Overall, 28% (1,608/5,771) of male participants reported any alcohol use, with 10% (567/5,771) meeting criteria for hazardous alcohol use (AUDIT-C score ≥4; Table 1). Unemployment was common in the cohort (65.7%; 3,256/5,771) and was higher among men with no/low risk alcohol use (67.9% versus 56.9% hazardous alcohol use; p-value<0.001). Participants with hazardous alcohol consumption were significantly more likely to report anxiety/depression (14.9% vs 9.0%, p-value<0.001), smoking (62.7% vs 17.6%, p-value<0.001), and belong to lower socioeconomic quintiles (p-value=0.003). Notable differences were observed in care cascades for HIV and diabetes across alcohol use groups, whereas no significant differences were found for tuberculosis or hypertension care cascades ( Table 1).

**Table 1.** Characteristics of Men by Hazardous Alcohol Use Thresholds.

Characteristic	No/low risk alcohol use (n=5,204)	Hazardous alcohol use (n=567)	Total (n=5,771)	p-value
**Age (years)**	35.7 (SD 19.2)	40.6 (SD 14.7)	36.2 (18.9)	<0.001
**Highest Level Education**				
No schooling	513 (9.9)	69 (12.3)	582 (10.1)	0.005
Primary Grades 1–8	480 (9.3)	67 (11.9)	547 (9.5)	
High School	4,188 (80.8)	427 (75.8)	4,613 (80.3)	
**Ever smoker**	914 (17.6)	358 (63.1)	1,272 (22.0)	<0.001
**Employed**	1,089 (32.1)	146 (43.1)	1,707 (34.3)	<0.001
**Socioeconomic status**				
Lowest	590 (11.6)	88 (15.7)	678 (12.0)	0.003
Low	1,330 (26.2)	161 (28.9)	1,491 (26.5)	
Middle	1,202 (23.7)	135 (24.1)	1,337 (23.8)	
High	913 (18.0)	91 (16.3)	1,003 (17.8)	
Highest	1,035 (20.4)	85 (15.2)	1,120 (19.9)	
**Anxiety/depression**	405 (9.0)	60 (14.9)	465 (9.4)	<0.001
**Hypertension Cascade**				
No hypertension	4,279 (82.0)	464 (81.6)	4,743 (82.0)	0.973
Hypertension, not in care	478 (9.2)	65 (11.4)	543 (9.4)	
Hypertension, uncontrolled in care	187 (3.6)	21 (3.7)	208 (3.6)	
Hypertension controlled	272 (5.2)	19 (3.3)	291 (5.0)	
**Diabetes Cascade**				
No Diabetes	4,900 (94.2)	555 (98.1)	5,455 (94.5)	<0.001
Diabetes, not in care	245 (4.7)	11 (1.9)	256 (4.4)	
Diabetes uncontrolled in care	30 (0.6)	0 (0)	30 (0.5)	
Diabetes controlled	29 (0.6)	0 (0)	29 (0.5)	
**Tuberculosis Cascade**				
No active TB	3,009 (96.9)	347 (96.4)	3,356 (96.8)	0.646
Active TB, not on treatment	70 (2.3)	10 (2.8)	80 (2.3)	
On TB treatment	28 (0.9)	3 (0.8)	31 (0.9)	
**HIV Cascade**				
HIV Negative	3,978 (76.4)	387 (68.3)	4,365 (75.6)	<0.001
Unaware living with HIV	243 (4.7)	33 (5.8)	276 (4.8)	
Diagnosed, not in care	48 (0.9)	18 (3.2)	66 (1.1)	
In care, not controlled	100 (1.9)	15 (2.7)	115 (2.0)	
HIV disease controlled	835 (16.1)	114 (20.1)	949 (16.4)	

Values are numbers (percentage) Age (years) is mean (standard deviation) p<0.05 for comparisons between alcohol consumption groups by Kruskal-Wallis testing

### Association between alcohol use and HIV-clinical outcomes among men

**Figure 2.**
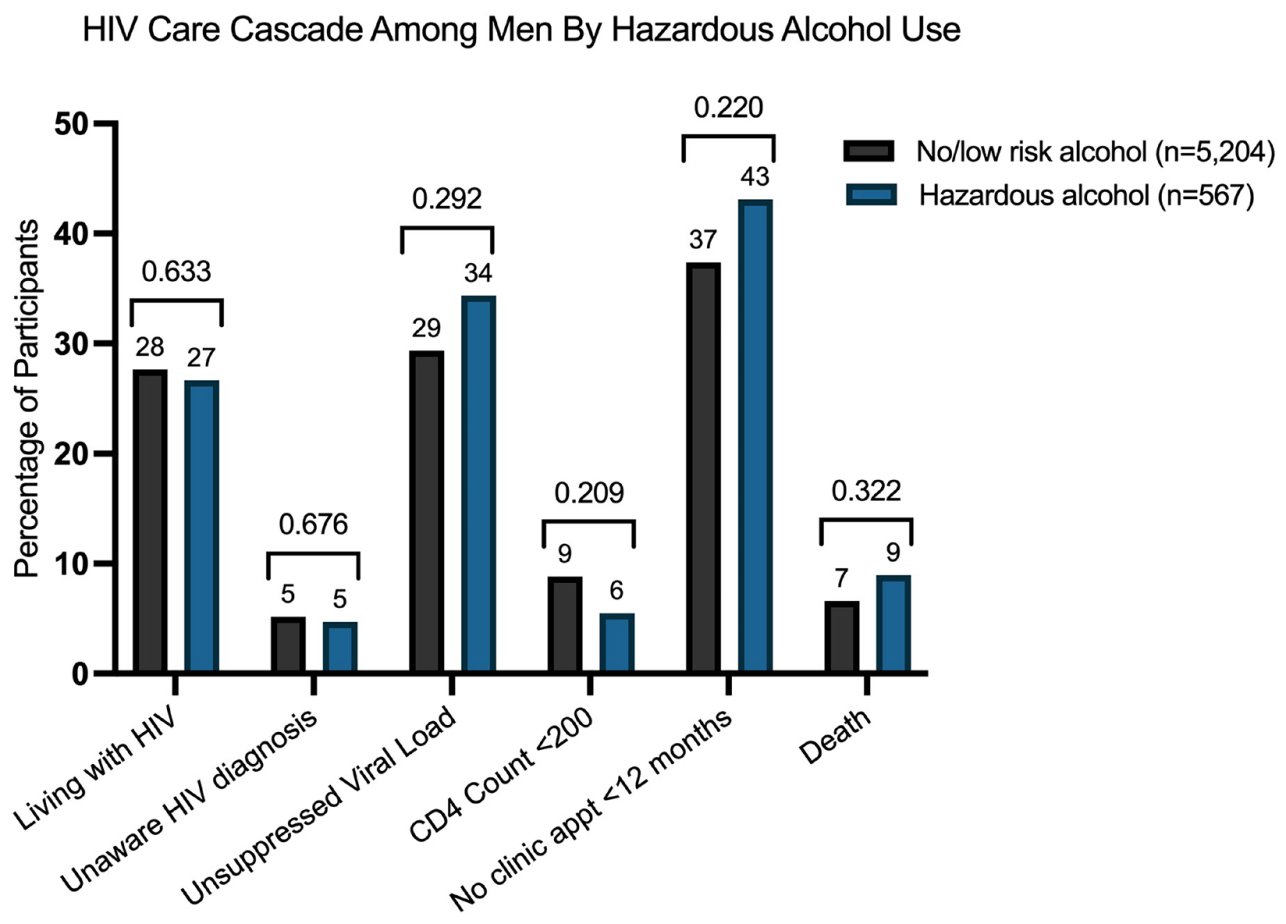
Adjusted Prevalences of HIV Care Cascade Outcomes among men by hazardous alcohol. This figure displays the adjusted prevalence ratios (aPRs) for the association between hazardous alcohol use and key HIV care cascade outcomes: HIV prevalence, unawareness of HIV diagnosis, unsuppressed viral load, advanced HIV disease (CD4<200), no clinic appointment within 12 months, and death following the health screening.

Among the men in our analytic cohort, 24% (1406/5771) were living with HIV, with a higher prevalence observed in those reporting hazardous alcohol use compared to who did not (32% versus 24%, respectively, p-value<0.001) ( Table 2). In univariate analysis, men with hazardous alcohol use had a significantly higher prevalence of HIV compared to those without (PR 1.35, 95% CI: 1.18–1.53; p < 0.001) ( Table 2). However, this association was not significant after adjusting for demographic and clinical confounders (aPR 0.96, 95% CI: 0.83–1.12; p = 0.633; Figure 2). Additional factors independently associated with living with HIV in multivariable models included older age, lower socioeconomic status, high school completion, and being employed ( Table 2). At the time of health screening, 6% (33/567) of men reporting hazardous alcohol use and 5% (243/5,204) of those with no/low risk alcohol use, were unaware of their HIV-positive status (p-value=0.223) ( Table 2). Hazardous alcohol use was not associated with awareness of a new HIV diagnosis at health screening, as shown in both univariate (PR 1.25, 95% CI 0.88–1.77; p-value=0.222) and multivariate models (aPR 0.91, 95%CI 0.58–1.42; p-value-0.676) ( Table 2, Figure 2).

**Table 2.** Poisson Regression Models for HIV serostatus and unknown HIV diagnoses among men.

Characteristic	Crude Prevalence	Unadjusted Prevalence Ratio (95%CI)	p-value	Adjusted Prevalence Ratio (95%CI)	p-value
2A. Model Outcome: Men Living with HIV (n=5,771)					
No/low risk alcohol	1,226/5,204 (24%)	Reference group			
Hazardous alcohol	180/567(32%)	1.35 (1.18-1.53)	<0.001	0.96 (0.83-1.12)	0.633
Age 15–24 years	142/2,199 (10%)	Reference group			
Age 25–34 years	312/1,050 (22%)	4.60 (3.83- 5.53)	<0.001	4.03 (3.17-5.13)	<0.001
Age 35–49 years	585/1,061 (42%)	8.54 (7.22-10.10)	<0.001	7.50 (5.96-9.42)	<0.001
Age 50+ years	367/1,461 (26%)	3.89 (3.24-4.67)	<0.001	3.88 (3.02-4.98)	<0.001
Socioeconomic status score (mean 0.25, SD 2)	0.11 (SD 2)	0.97 (0.94-0.99)	0.003	0.91 (0.88-0.95)	<0.001
No Anxiety/Depression	1,067/4,445 (24%)	Reference group			
Anxiety/Depression	149/462 (32%)	1.34 (1.17-1.55)	0.083	1.09 (0.95-1.26)	0.218
*Max education level* None	143/579 (25%)	Reference group			
*Max education level* Primary Grade 1–8	144/546 (26%)	1.07 (0.87-1.30)	0.519	1.03 (0.83-1.27)	0.797
*Max education level* High School	1,095/4,590 (24%)	0.97 (0.83-1.12)	0.653	1.29 (1.07-1.54)	0.006
Unemployed	730/3,256 (22%)	Reference group			
Employed	631/1,698 (37%)	1.66 (1.52-1.81)	<0.001	1.13 (1.02-1.25)	0.016
2B. Model Outcome: New HIV diagnosis among men (n=5,771)					
No/low risk alcohol	243/5,204 (5%)	Reference group			
Hazardous alcohol	33/567(6%)	1.25 (0.88-1.77)	0.222	0.91 (0.58-1.42)	0.676
Age 15–24 years	63/2,199 (3%)	Reference Group			
Age 25–34 years	109/1,050 (10%)	3.62 (2.68-4.90)	<0.001	3.05 (2.03- 4.59)	<0.001
Age 35–49 years	69/1,061 (7%)	2.27 (1.63-3.17)	<0.001	2.00 (1.28-3.12)	0.002
Age 50+ years	35/1,461 (2%)	0.84 (0.56-1.26)	0.390	0.79 (0.44-1.44)	0.446
Socioeconomic status score (mean 0.25, SD 2)	0.28 (SD 2)	1.01 (0.95- 1.06)	0.771	0.98 (0.91 - 1.05)	0.511
No Anxiety/Depression	223/4,445 (5%)	Reference group			
Anxiety/Depression	16/462 (3%)	0.69 (0.42-1.14)	0.145	0.86 (0.50-1.48)	0.582
*Max education level* None	14/579 (2%)	Reference group			
*Max education level* Primary Grade 1–8	14/546 (3%)	1.06 (0.51-2.20)	0.875	0.85 (0.36-1.99)	0.707
*Max education level* High School	244/4,590 (5%)	2.20 (1.29-3.74)	0.004	1.37 (0.66 – 2.86)	0.397
Unemployed	136/3,256 (4%)	Reference group			
Employed	122/1,698 (7%)	1.72 (1.36-2.18)	<0.001	1.18 (0.88- 1.59)	0.267

### Association between alcohol use and HIV clinical outcomes among men living with HIV

All men living with HIV in the cohort completed viral load testing (1,406/1,406) and 99% (1,392/1,406) underwent CD4 cell count analyses. Overall, 70% of men living with HIV achieved viral suppression. The individuals with suppressed viral loads were similar between men with and without hazardous alcohol use (70% vs. 64%, p-value=0.119) ( Table 3). In univariate and multivariate analyses, hazardous alcohol use was not associated with viral suppression (aPR 0.93, 95%CI 0.81–1.07, p-value=0.292) ( Table 3, Figure 2). Among men living with HIV, 9% had evidence of advanced HIV disease with CD4 lymphocytes <200 cells/mL, however this did not differ between those with or without hazardous alcohol use (7% vs. 9% p-value=0.219). ( Table 3). This lack of association was consistent in both univariate and multivariate analyses ( Table 3, Figure 2).

**Table 3.** Poisson Regression models for HIV clinical outcomes among men living with HIV.

Characteristic	Crude Prevalence	Unadjusted Prevalence Ratio (95%CI)	p-value	Adjusted Prevalence Ratio (95%CI)	p-value
3A. Model Outcome: Men with viral suppression (n=1,406)					
No/low risk alcohol	862/1,226 (70%)	Reference group			
Hazardous alcohol	116/180 (64%)	0.92 (0.82-1.03)	0.136	0.93 (0.81-1.07)	0.292
Age 15–24 years	72/142 (51%)	Reference group			
Age 25–34 years	158/312 (51%)	1.00 (0.82-1.21)	0.990	1.22 (0.93-1.62)	0.157
Age 35–49 years	430/585 (74%)	1.45 (1.22-1.72)	<0.001	1.75 (1.35-2.27)	<0.001
Age 50+ years	318/367 (87%)	1.71 (1.45-2.02)	<0.001	1.95 (1.50-2.53)	<0.001
Socioeconomic status score (mean 0.25, SD 2)	0.07 (SD 2)	0.99 (0.97-1.01)	0.344	1.01 (0.99-1.03)	0.484
No Anxiety/Depression	731/1,067 (69%)	Reference group			
Anxiety/Depression	116/149 (78%)	1.14 (1.03-1.25)	0.008	0.99 (0.90-1.09)	0.856
*Max education level* None	123/146(86%)	Reference group			
*Max education level* Primary Grade 1–8	118/144 (82%)	0.95 (0.86-1.05)	0.348	0.94 (0.85-1.05)	0.273
*Max education level* High School	719/1,095 (66%)	0.76 (0.71-0.83)	<0.001	0.90 (0.82-0.98)	0.015
Unemployed	530/730 (73%)	Reference group			
Employed	421/631 (67%)	0.92 (0.86-0.99)	0.019	0.95 (0.88-1.02)	0.164
3B. Model Outcome: CD4 lymphocytes <200 cells/mm3 in men (n=1,392)					
No/low risk alcohol	115/1,213 (9%)	Reference group			
Hazardous alcohol	12/179 (7%)	0.71 (0.40-1.26)	0.236	0.62 (0.30- 1.30)	0.209
Age 15–24 years	11/140 (8%)	Reference group			
Age 25–34 years	38/308 (12%)	1.57 (0.83-2.98)	0.168	1.35 (0.61-2.98)	0.459
Age 35–49 years	65/581 (11%)	1.42 (0.77-2.63)	0.258	1.30 (0.60-2.80)	0.510
Age 50+ years	13/363 (4%)	0.46 (0.21-0.99)	0.048	0.49 (0.18-1.28)	0.143
Socioeconomic status score (mean 0.25, SD 2)	0.24 (SD 2)	1.03 (0.94-1.13)	0.481	1.00 (0.89-1.12)	0.947
No Anxiety/Depression	99/1,060 (9%)	Reference group			
Anxiety/Depression	6/148 (4%)	0.43 (0.19-0.97)	0.042	0.53 (0.22-1.27)	0.170
*Max education level* None	7/142 (5%)	Reference group			
*Max education level* Primary Grade 1–8	7/142 (5%)	1.00 (0.36-2.78)	1.00	1.60 (0.41-6.24)	0.496
*Max education level* High School	112/1,084(10%)	2.10 (1.00-4.41)	0.051	2.37 (0.74-7.61)	0.148
Unemployed	62/722 (9%)	Reference group			
Employed	62/625 (10%)	1.15 (0.83-1.62)	0.399	0.98 (0.66-1.46)	0.938

### Association between alcohol use and retention in clinic among men living with HIV

**Figure 3.**
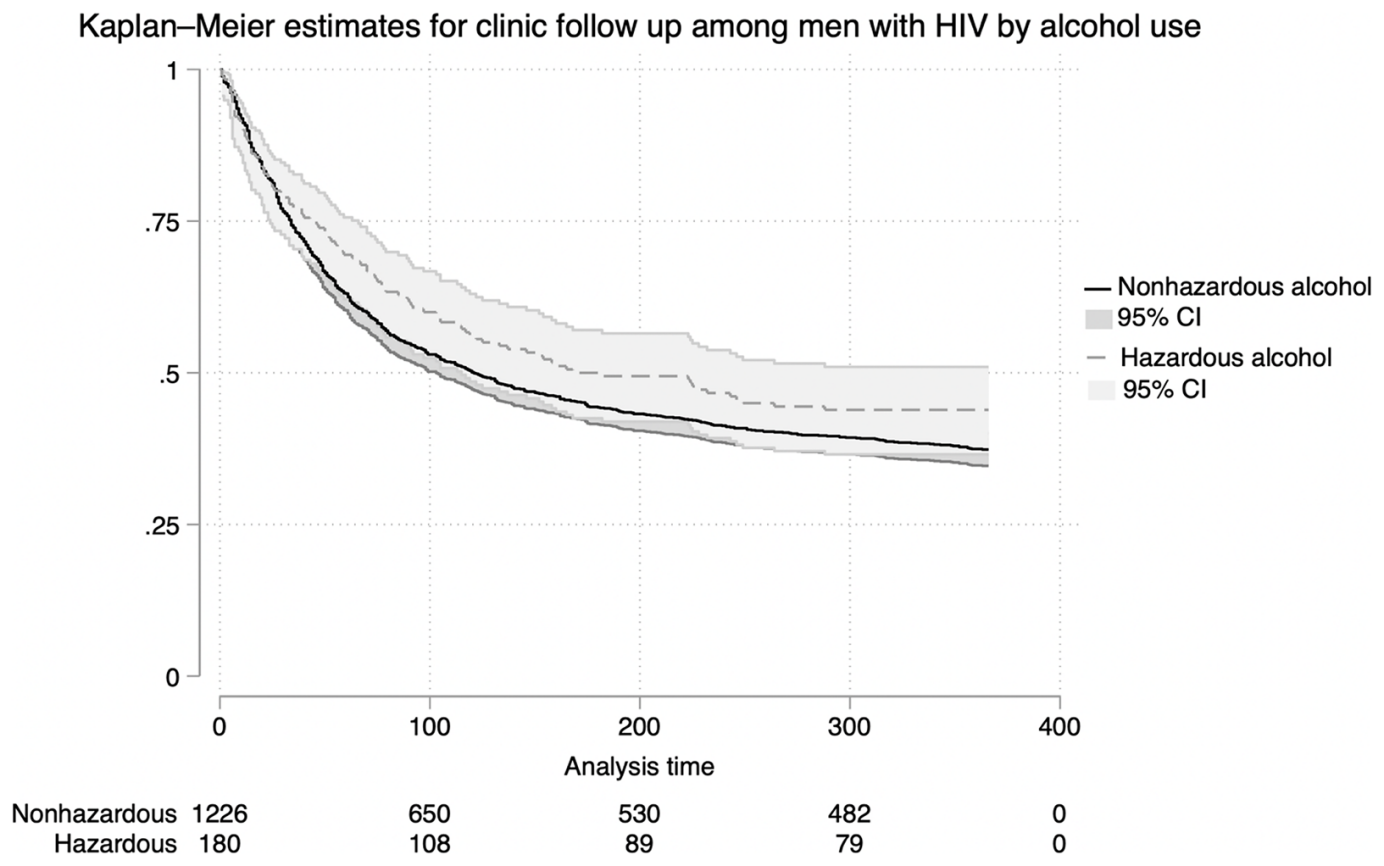
Kaplan-Meier Estimates for Clinic Retention Among Men Living with HIV. Kaplan-Meier survival curves comparing time to clinic visit after health screening among men with hazardous versus no/low-risk alcohol use.

Among the 1,406 men living with HIV, 63% (768/1,226) of those with no/low risk alcohol use attended the clinic for follow-up within 12 months after the Vukuzazi study, compared to 56% (101/180) of men with hazardous alcohol consumption (p = 0.092) ( Table 4). Kaplan-Meier survival curves were generated to compare the time to clinic follow-up among men with hazardous alcohol use versus those with no/low risk alcohol ( Figure 3). Men with hazardous consumption had a longer median time to clinic follow-up at 182 days compared to 121 days for men with no/low risk alcohol use, however this was not significant by log-rank testing (p = 0.099).

**Table 4.** Poisson Regression Model for clinic retention among men living with HIV.

Characteristic	Crude Prevalence	Unadjusted Rate Ratio (95%CI)	p-value	Adjusted Rate Ratio (95%CI)	p-value
Model Outcome: Retention in clinic within 12 months following screening (n=1,406)					
No/low risk alcohol	768/1,226 (63%)	Reference group			
Hazardous alcohol	101/180 (56%)	0.90 (0.78-1.03)	0.113	0.91 (0.78-1.06)	0.220
No New Diagnoses	656/967 (68%)	Reference group			
New Diagnosis*	213/439 (49%)	0.72 (0.64-0.79)	<0.001	0.75 (0.67-0.85)	<0.001
Age 15–24 years	79/142 (56%)	Reference group			
Age 25–34 years	139/312 (45%)	0.80 (0.66-0.97)	0.023	0.99 (0.76-1.29)	0.926
Age 35–49 years	382/585 (65%)	1.17 (1.00-1.38)	0.047	1.39 (1.09-1.77)	0.009
Age 50+ years	269/367 (73%)	1.32 (1.12-1.55)	0.001	1.47 (1.15-1.89)	0.002
Socioeconomic status score (mean 0.25, SD 2)	-0.02 (SD 2)	0.97 (0.95-0.99)	0.003	0.98 (0.96-1.01)	0.161
No Anxiety/Depression	651/1,067 (61%)	Reference group			
Anxiety/Depression	105/149 (70%)	1.16 (1.03-1.30)	0.014	1.03 (0.91-1.16)	0.872
*Max education level* None	105/143 (74%)	Reference group			
*Max education level* Primary Grade 1–8	100/144 (69%)	0.95 (0.82-1.10)	0.456	0.99 (0.85-1.15)	0.872
*Max education level* High School	648/1,095 (59%)	0.81 (0.72-0.90)	<0.001	0.99 (0.87-1.13)	0.938
Unemployed	478/730 (65%)	Reference group			
Employed	359/631 (57%)	0.87 (0.80-0.95)	0.001	0.88 (0.80-0.97)	0.008

In unadjusted and adjusted analyses, hazardous alcohol use was not associated with retention in clinic ( Table 4, Figure 2). Conversely, men with any new diagnosis (HIV, TB, diabetes, hypertension) at the health screening were significantly less likely to attend follow-up visits compared to those without a new diagnosis. This association is observed in both univariate (PR 0.72, 95%CI 0.64–0.79, p-value < 0.001) and adjusted models (aRR 0.75, 95%CI 0.67–0.85, p-value < 0.001). Additionally, being employed was independently associated with a lower likelihood of attending appointments at public healthcare clinics in multivariate models (aRR 0.88, 95%CI 0.80–0.97, p-value=0.008) ( Table 4).

### Association between alcohol use and all-cause mortality among men living with HIV

Nearly all men in this analysis (99.9%, 5766/5771) were accounted for in the surveillance system after the Vukuzazi health screening up until August 2023. The mean follow-up time was 3.4 years (SD 1.2 years), with a maximum follow-up time of 5.0 years. Among the participants reached, 316 men died (5.5%), 78 out-migrated (1.4%), and 93 were lost to follow-up (1.6%). Rates of out-migration (1.3% vs. 2.1%) and loss to follow-up (1.5% vs. 2.3%) were similar between alcohol use groups.

Among men living with HIV, 99.9% (1,404/1,406) had follow-up data, which included 102 deaths (7.3%), 24 out-migrations (1.7%), and 30 individuals lost to follow-up (2.1%). There were no significant differences in mortality (7% hazardous alcohol vs 7% no/low-risk alcohol, p-value=0.524, Table 5), outmigration, or lost to follow up by alcohol use groups.

**Table 5.** Cox Proportional Hazard Models for death among men with HIV.

Characteristic	Crude Prevalence	Unadjusted Hazard Ratio (95%CI)	p-value	Adjusted Hazard Ratio (95%CI)	p-value
Model Outcome: Death (n=1,404)					
No/low risk alcohol	89/1,224 (7%)	Reference group			
Hazardous alcohol	13/180 (7%)	0.98 (0.55-1.76)	0.954	1.41 (0.72 – 2.76)	0.322
Age 15–24 years	4/141 (3%)	Reference group			
Age 25–34 years	13/312 (4%)	1.45 (0.47-4.44)	0.518	2.88 (0.36-22.90)	0.318
Age 35–49 years	36/584 (6%)	2.21 (0.79-6.20)	0.133	5.15 (0.69-38.32)	0.109
Age 50+ years	49/367 (13%)	5.01 (1.81-13.88)	0.002	7.78 (1.03-58.75)	0.047
Socioeconomic status score (mean 0.25, SD 2)	0.60 (SD 1.8)	1.13 (1.02 – 1.25)	0.016	1.15 (1.03 - 1.29)	0.011
No Anxiety/Depression	61/1,083 (5%)	Reference group			
Anxiety/Depression	23/152 (15%)	2.85 (1.76 - 4.61)	<0.001	1.87 (1.10 – 3.19)	0.022
*Max education level* None	20/143 (14%)	Reference group			
*Max education level* Primary Grade 1–8	12/143 (8%)	0.58 (0.28 -1.18)	0.134	0.66 (0.30 – 1.46)	0.309
*Max education level* High School	67/1,095 (6%)	0.41 (0.25 - 0.68)	<0.001	0.63 (0.33 – 1.17)	0.144
Unemployed	64/730 (9%)	Reference group			
Employed	35/630 (6%)	0.62 (0.41 - 0.94)	0.024	0.68 (0.41 - 1.12)	0.132

**Figure 4.**
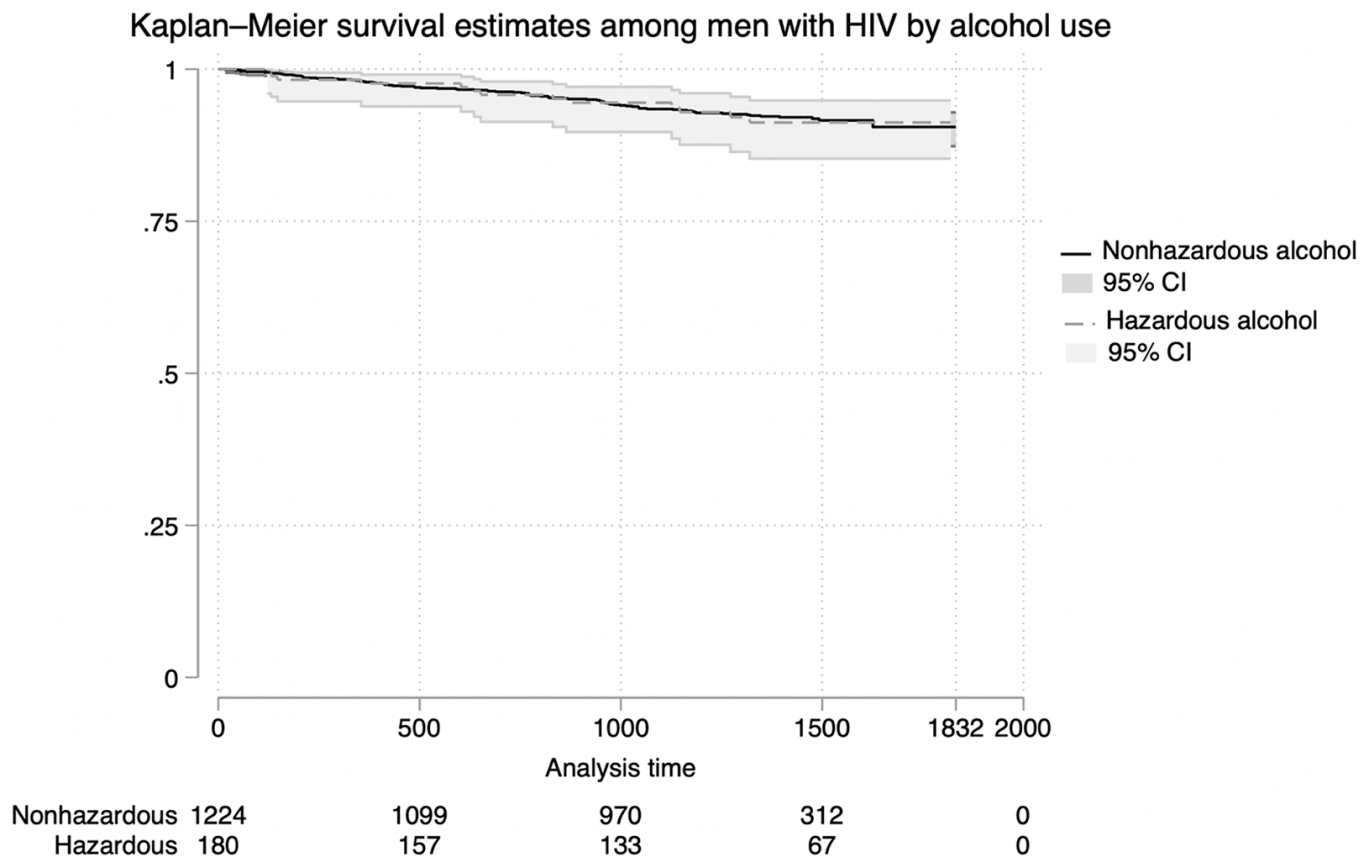
Kaplan-Meier Survival Estimates for All-Cause Mortality Among Men Living with HIV. Kaplan-Meier survival curves comparing time to death among men living with HIV stratified by hazardous alcohol use. No significant differences in mortality are observed between alcohol use groups over the follow-up period.

Kaplan-Meier survival curves were used to compare time to death between men with and without hazardous alcohol use ( Figure 4). No significant differences in survival were observed between groups (log-rank test, p-value = 0.925). In both unadjusted and adjusted models, hazardous alcohol use was not associated with mortality (aHR 1.41, 95% CI 0.72 – 2.76, p-value=0.322) ( Table 5, Figure 2). Conversely, higher socioeconomic status, age 50 years and older, and the presence of anxiety or depression were independently associated with an increased risk of death in multivariate models ( Table 5). The proportional hazards assumption was assessed using Schoenfeld residuals and was not violated for any variable (global test, p = 0.3572).

In sensitivity analyses redefining alcohol use as any alcohol consumption in the past 12 months, there was no association between alcohol use and HIV serostatus (aPR 1.05, 95% CI 0.95–1.16, p-value=0.352) ( **Table S1**) (extended data). Similar to the primary analyses, alcohol use was not significantly associated with virologic suppression, advanced HIV disease, clinical access to care, or mortality ( **Tables S2-S4**) (extended data).

## Discussion

In a large, well-characterized population from a rural, HIV-endemic region of South Africa, we found that hazardous alcohol use among men was not associated with HIV prevalence after adjusting for confounders. This contrasts with prior studies in KwaZulu-Natal, which identified hazardous alcohol use as a common factor among men at risk for acquiring HIV, regardless of marital or employment status ^31^. Contrary to our initial hypothesis, alcohol use was also not associated with virologic suppression or the presence of advanced HIV disease suggesting that hazardous alcohol use may not impair clinical outcomes among men living with HIV in this setting. While retention in clinical care within one year of the health screening was suboptimal across the region's 11 public health clinics, hazardous alcohol use did not significantly impact attendance rates, nor was it associated with short-term mortality. These findings challenge the assumption that hazardous alcohol use leads to poorer HIV-related clinical outcomes. Instead, they may reflect the benefits of South Africa’s evolving HIV care landscape, including modern antiretroviral regimens and differentiated service delivery models, such as the Central Chronic Medicine Dispensing and Distribution (CCMDD) program ^32, 33^. In particular, once-daily, integrase inhibitor-based ART regimens may offer resilience against intermittent adherence due to alcohol use, sustaining viral suppression even in the context of binge drinking or weekend alcohol use ^34^. Taken together, our findings support a shift away from stigmatizing men living with HIV who use alcohol, and toward a more nuanced understanding of how community-based, patient-centered approaches can better meet the needs of this population ^35^.

The prevalence of both hazardous (10%) and any alcohol use among men in our cohort (28%) was notably lower than rates reported in national surveys and other regional studies. For example, the 2020 Global Burden of Disease Study reported an alcohol use prevalence of 47.4% among South African men aged 15–39 years ^15^. Additionally, within the same Health and Demographic Surveillance System where our study was conducted, a study among a random sampling of young men found recent alcohol use to be 57% among the cohort, with 45% meeting criteria for hazardous use ^36^. The lower prevalence of alcohol use in our cohort may reflect a selection bias, as older individuals and those proactive about health screening could have been more likely to participate in the community-based health screening. Furthermore, our study was conducted in the context of universal HIV testing and treatment, which may have contributed to the observed clinical outcomes. Hazardous alcohol use was not associated with HIV prevalence, viral load suppression, nor advanced HIV disease once controlled for other social determinants of health. This aligns with results from a multinational cohort of people living with HIV in Uganda, Russia, and the United States, where severe alcohol use disorder did not impair HIV viral suppression or CD4 count ^32^. The authors emphasized that people living with HIV and severe alcohol use disorder in the current era of antiretroviral therapy can achieve virologic control ^32^. Similarly, the SEARCH trial in Kenya and Uganda found that universal test-and-treat combined with patient-centered care led to high levels of ART initiation and viral suppression among individuals reporting hazardous alcohol use ^35^. These results are consistent with what we observed in our rural South African cohort.

Two key factors associated with alcohol use and health outcomes were employment and mental health, particularly self-reported anxiety or depression. Employment emerged as a significant factor associated with living with HIV and lower rates of public clinic engagement in care. While employment may contribute to stress and increased alcohol consumption, it also provides greater financial access to alcohol. For example, a study on substance use in various occupations in sub-Saharan Africa identified disposable income and poverty as key drivers of substance use ^37^. Another South African study found that for each percentage increase in income spent on alcohol, there was a significant rise in the biomarker phosphatidylethanol (PEth), translating to a 14% higher risk of unhealthy alcohol use ^38^. These findings suggest that employment may amplify alcohol use by providing disposable income, which in turn may enable behaviors that increase HIV transmission.

Anxiety and depression were associated with an increased risk of short-term mortality and were more prevalent among men with hazardous alcohol consumption. Studies in sub-Saharan Africa have highlighted severe stress, anxiety, and depression as significant contributors to increased alcohol use ^38– 40^. One South African study outside informal alcohol-serving venues reported that individuals living with HIV experienced higher perceived life stress than those without HIV ^40^. This stress was linked to more drinking days, intoxication, and tavern visits, leading researchers to conclude that stress mediates the relationship between HIV status and alcohol use ^40^. In our study, the association between anxiety/depression and short-term mortality may reflect a bidirectional relationship: individuals with anxiety or depression could have underlying health conditions contributing to mortality risk, or anxiety and depression may augment the impact of alcohol use on mortality through poorer health behaviors or delayed care-seeking.

While alcohol use was not associated with clinic retention within 12 months, the overall low rates of healthcare engagement (56% among hazardous alcohol group and 63% among no/low-risk alcohol) is concerning. These findings are consistent with studies highlighting systemic barriers to healthcare among men in South Africa, including stigma, competing economic priorities, structural challenges such as limited clinic accessibility, and power dynamics with healthcare providers ^41^. For example, a qualitative study in Tshwane, South Africa reported that provider-patient power imbalances discouraged men living with HIV from discussing challenges related to alcohol use and ART adherence ^41^. The authors recommended patient-centered approaches to foster greater involvement of men in their treatment plans ^41^. Interestingly, our results show that a new diagnosis during the community-based health screening was associated with a lower adjusted rate ratio of retention at public healthcare clinics. Alcohol use has been linked to both HIV-related and non-HIV-related multimorbidity in rural South African populations ^42^. Yet, when diagnosed, men appear less likely to attend clinics for follow-up care. We suspect this may reflect a preference for privative healthcare services, driven by perceptions of convenience and better quality of care – an observation supported by prior studies ^43^.

In contrast to prior studies, we found no significant association between alcohol use and mortality. Probst *et al.* previously reported that alcohol use significantly increased HIV-related mortality, particularly among men of lower socioeconomic status, and contributed to socioeconomic disparities in HIV-related outcomes ^22^. Interestingly, our study found that higher socioeconomic status was associated with greater mortality in adjusted analyses, a finding that contradicts existing literature ^22^. This may be explained by factors such as employment, which provides disposable income for alcohol consumption, fast food, and private transportation—behaviors that may increase socioeconomic status while also elevating mortality risk. Furthermore, the lack of an observed mortality association with alcohol excess in our study could reflect the relatively young age of our cohort and the shorter follow-up period, which may limit the ability to detect long-term health impacts.

Our findings emphasize the importance of integrated approaches that address alcohol use, even when direct associations between alcohol use and HIV clinical outcomes are not observed. Emerging evidence suggests that even moderate alcohol consumption is associated with elevated risks for cardiovascular disease, cancer, and mental health conditions ^44– 46^. Therefore, incorporating harm-reduction strategies, such as motivational interviewing, alcohol cessation interventions, and screening for mental health comorbidities ^47, 48^, could provide substantial public health benefit. Tailored, community-based interventions, such as peer support programs and mobile clinics in settings that serve alcohol, could improve the accessibility of substance use care for men.

The strengths of our study include a large, population-based cohort that was thoroughly characterized with testing for HIV serostatus, viral load, and CD4 count. Additionally, the longitudinal follow-up from a health and demographic surveillance system allowed for assessment of public health clinic visits and mortality outcomes. There were also important limitations. The cross-sectional design of the alcohol use measures, HIV serostatus, viral load, and CD4 count precludes causal inferences between alcohol use and HIV-related clinical outcomes. As such, although they were correlated, we cannot be sure that hazardous alcohol use predates HIV acquisition or that another factor (e.g. mental health disorder) was responsible for both. Moreover, the AUDIT-C questionnaire deviated from the standard format by (1) capturing monthly alcohol consumption instead of the typical 12-month timeframe and (2) requiring extrapolation or mapping of responses to align with AUDIT-C scoring, which may have affected the validity of the exposure variable. The reliance on self-reported data for alcohol consumption introduces the potential for recall and social desirability bias, although we did not see any differences in outcomes when examining any alcohol use. Lastly, the lack of data on private healthcare engagement within the Health and Demographic Surveillance System may have biased the clinic visit outcomes.

## Conclusion

Our study demonstrated that hazardous alcohol use among men in a rural, HIV-endemic region of South Africa does not affect HIV prevalence, virologic suppression, CD4 count, or short-term mortality in the era of modern ART regimens. Low clinic retention emphasizes the need for tailored, community-based interventions and harm-reduction strategies to better engage men with hazardous alcohol use.

## Declarations

### Ethics approval and consent to participate

The institutional review boards approved the Vukuzazi Study at the University of KwaZulu-Natal Biomedical Research Ethics Committee (BE560/17) and Mass General Brigham (Protocol 2018P001802). Furthermore, the Health and Demographic Surveillance System was approved at the University of KwaZulu-Natal Biomedical Research Ethics Committee (BE290/16). All participants gave written informed consent to participate. For adolescents aged 15–17 years, both written assent from the participant and written informed consent from a parent, legal guardian, or pragmatic parental substitute were obtained, in accordance with South African ethical guidelines. In cases where adolescents were considered emancipated minors (e.g., married, parents, or living in child-headed households), independent consent was permitted, with the circumstances documented.

### Consent for publication

Not applicable

## Data Availability

The derived dataset and accompanying data dictionary used for this analysis are publicly available through the Africa Health Research Institute (AHRI) Data Repository ( https://doi.org/10.23664/ahri.menalcoholmerged1-3) ^49^. The dataset within this repository meets open data standards, including the assignment of open licenses (CC-BY 4.0), provision of DOIs, no login requirements for access, and a commitment to long-term data preservation. The raw datasets used in the study contain sensitive identifiers and cannot be shared publicly, but are available through restricted-access requests; please email RDMServiceDesk@ahri.org. The three dataset include: 1) https://doi.org/10.23664/AHRI.VUKUZAZI.2021 ^50^; 2) https://doi.org/10.23664/AHRI.SEB ^51^; 3) https://doi.org/10.23664/AHRI.AHRILINK.Individual.Visits ^52^. Access to restricted datasets may be granted after publication, subject to approval of the proposed analyses by the Principal Investigator and the completion of a data access agreement. To protect participant confidentiality, only the derived, de-identified dataset will be shared openly. This study is a secondary analysis of previously collected data from the Vukuzazi study and the AHRI Health and Demographic Surveillance System (HDSS). No new data collection instruments (e.g., questionnaires, consent forms, interview guides) were developed for this analysis. Supplementary materials are shared in the publicly available datasets in the AHRI Data Repository ( https://doi.org/10.23664/ahri.menalcoholmerged1-3) ^49^.
