# Supplementary material for: Alcohol Consumption and Health Outcomes Among Men in a Rural HIV-Endemic Cohort in South Africa
Source: Wellcome Open Res. 2026 Jan 27;10:469. Originally published 2025 Aug 29. [Version 2] doi: 10.12688/wellcomeopenres.24185.2 (PMC13107110; doi:10.12688/wellcomeopenres.24185.2)
Supplement: Peer review history

# Reviewer report: Reviewer response for version 2

**DOI:** 10.21956/wellcomeopenres.28489.r152717

**Published:** 2026-04-23

**Authors:** Veysel Akca

**Affiliations:** 1 Muğla University, Muğla, Turkey

Thank you for your contribution to this important area of research. I found the manuscript to be very well written and methodologically sound. I have only a few minor comments that may help further improve the clarity and presentation of the work.

1. Viral suppression and laboratory outcomes appear relatively favorable in a rural cohort characterized by high levels of unemployment and socioeconomic disadvantage, which may reflect the effectiveness of modern ART regimens, relatively strong access to health services, and potential selection bias among participants engaged in community-based screening; therefore, this finding should be interpreted cautiously rather than suggesting that structural disadvantage has limited influence on HIV outcomes in this setting.
2. Lower reported alcohol use among unemployed individuals may be influenced not only by true behavioral differences but also by social desirability bias, reverse causality, and differential reporting accuracy across socioeconomic groups; these factors should be considered more explicitly when interpreting the observed associations.
3. The presentation of covariates across both the covariates and statistical analysis sections appears somewhat repetitive, and clarity could be improved by fully detailing confounders in the covariates section while limiting the statistical analysis section to a concise description of model adjustments.
4. The manuscript would benefit from a clearer discussion of statistical power, particularly given that the study does not appear to have been originally powered for all outcomes; a post-hoc power assessment or reporting of minimum detectable effect sizes (e.g., using PASS or G*Power) would help contextualize the interpretation of non-significant findings.
5. The concluding statement may be somewhat strong in implying a lack of association between alcohol use and HIV outcomes, and it may be preferable to adopt more cautious wording that acknowledges the observational design, potential residual confounding, and the multifactorial nature of HIV care engagement and adherence.
6. Although follow-up appears robust, potential differences in risk profiles among individuals who out-migrated from the surveillance area may introduce selection bias, which should be more explicitly acknowledged as a limitation affecting both mortality and retention estimates.
7. The relatively high viral suppression rates observed in this rural setting may reflect strong health system performance and ART accessibility, but may also be influenced by selection bias related to participation in community-based screening, which could limit generalizability.
8. The association between alcohol use and socioeconomic status may be affected by measurement bias, particularly social desirability bias, which could lead to underreporting of alcohol consumption and attenuation of observed associations.
9. The cross-sectional measurement of alcohol exposure limits causal inference regarding its relationship with HIV outcomes, and raises the possibility of reverse causality, as exposure and outcomes were assessed at the same time point.
10. The absence of key confounders such as ART adherence, stigma, healthcare access barriers, and partner-related factors may introduce residual confounding and should be acknowledged as a limitation affecting interpretation of the findings.
11. While high viral suppression rates are encouraging in a rural cohort, they may also reflect selection bias due to differential participation in health screening, which could limit the representativeness of the sample and affect generalizability.
12. The relatively short follow-up period and wide confidence intervals suggest that long-term effects of alcohol use on mortality may not have been fully captured, and therefore conclusions regarding the absence of an association should be interpreted with caution, highlighting the need for longer follow-up studies.

Is the work clearly and accurately presented and does it cite the current literature?

Yes

If applicable, is the statistical analysis and its interpretation appropriate?

Yes

Are all the source data underlying the results available to ensure full reproducibility?

No source data required

Is the study design appropriate and is the work technically sound?

Yes

Are the conclusions drawn adequately supported by the results?

Yes

Are sufficient details of methods and analysis provided to allow replication by others?

Yes

Reviewer Expertise:

My research expertise includes HIV epidemiology, infectious diseases and population-based cohort studies.

I confirm that I have read this submission and believe that I have an appropriate level of expertise to confirm that it is of an acceptable scientific standard.

# Reviewer report: Reviewer response for version 1

**DOI:** 10.21956/wellcomeopenres.26678.r133826

**Published:** 2025-10-09

**Authors:** Gloria Guevara Alvarez

**Affiliations:** 1 New York University, New York, USA

Thank you for your contribution to this important area of research. I found the article was very well written. I have only minor comments.

 1) In the second to last paragrph of the introduction, you discuss integrated datasets provide a unique opportunity to evaluate men’s alcohol use with HIV-related health outcomes, healthcare engagement, and all-cause mortality in this high-burden region. 

 Can you elaborate more on the novelty of this study as it relates to alcohol research in this population , what is new about this study, why focus on alcohol based on the literature/

 Given the findings providing a strong rationale for why this study focused on alcohol would be beneficial ?

 2) For this sentence: Overall, fewer than two-thirds of men stayed connected to healthcare after screening, regardless of whether they drank alcohol.

 Please clarify if is this for the binary variable any alcohol or no alcohol or the variable the AUDIT categorical variable of AUDIT equal to or greater than 4?

 3) Two sections, covariates and stats analysis, seem to repeat information about covariates, I'd keep them in the covariate section and not repeat them in hte analysis section.

 "Potential confounders of the association between alcohol use and HIV clinical outcomes, clinic retention, and mortality included age, socioeconomic status, education level, employment status, and anxiety/depression. "

 "The following covariates were included: age, socioeconomic status, education level, employment status, and anxiety/depression. Smoking demonstrated collinearity with alcohol use and was not included in the final models."

 4) Are you able to provide information about what type of power your sample size provides? I recall using the PASS software to work backwards from the total number of participants in an analytical sample to establish what type of power could be attributed to the sample size of a study even if a sample size was not calculated inititally.

 5) the last sentence of the paper seems like an overreach:

 "These findings suggest that while alcohol use is common, it may not directly impact HIV outcomes."

 Evidence suggests there is a direct impact even when ART is adhered to. Perhaps you might comment that this study suggests that future work should look at mediators, and recommend mediation studies that unpack the mechanisms by which alcohol does or does not impact HIV outcomes in this population should be carried out.

Is the work clearly and accurately presented and does it cite the current literature?

Yes

If applicable, is the statistical analysis and its interpretation appropriate?

Yes

Are all the source data underlying the results available to ensure full reproducibility?

No source data required

Is the study design appropriate and is the work technically sound?

Yes

Are the conclusions drawn adequately supported by the results?

Yes

Are sufficient details of methods and analysis provided to allow replication by others?

Yes

Reviewer Expertise:

global health; integration of health services into HIV care including substance use. My region of work has largely been Sub Saharan Africa.

I confirm that I have read this submission and believe that I have an appropriate level of expertise to confirm that it is of an acceptable scientific standard.

# Response

**Published:** 2026-01-15

**Authors:** Alison Castle

**Affiliations:** Africa Health Research Institute, Durban, KwaZulu-Natal, South Africa

We thank the reviewer for their thoughtful and constructive feedback, and for their positive assessment of the manuscript. We have addressed each comment in detail below and revised the manuscript accordingly.

**Comment 1:** In the second to last paragraph of the introduction, please elaborate on the novelty of this study as it relates to alcohol research in this population. What is new about this study, and why focus on alcohol based on the literature? Given the findings, providing a stronger rationale for focusing on alcohol would be beneficial *.*

**Response: **Thank you for this suggestion. We revised the 2 ^nd^ to last paragraph of the introduction to clarify the novelty of this analysis, noting that much of the existing alcohol–HIV literature is based on clinic-based or cross-sectional studies conducted prior to the widespread use of integrase inhibitor–based ART. We highlight that because these regimens (TLD) achieve rapid viral suppression and have higher barriers to resistance, the clinical consequences of intermittent nonadherence associated with alcohol use may differ in the current ART era. The novelty of this study lies in leveraging linked, population-based longitudinal data from a setting where modern ART has been widely rolled out. We also strengthened the rationale for focusing on alcohol by expanding the preceding paragraph to more clearly situate hazardous alcohol use, based on the literature, as a driver of elevated HIV risk, delayed care engagement, and poorer health outcomes among men.

**Comment 2: **For the sentence: “Overall, fewer than two-thirds of men stayed connected to healthcare after screening, regardless of whether they drank alcohol.” Please clarify whether this refers to the binary any alcohol variable or the AUDIT categorical variable.

**Response: **Thank you for noting this ambiguity. We have clarified that this finding was consistent across both alcohol exposure definitions. Specifically, fewer than two-thirds of men attended a clinic visit within 12 months after screening regardless of whether alcohol use was defined as hazardous alcohol use (AUDIT-C ≥4) or as any alcohol consumption in the past 12 months.

**Comment 3:** The covariates and statistical analysis sections repeat information about covariates. Please keep these details in the covariates section and avoid repetition in the analysis section.

**Response:** The full description and justification of covariates now appears only in the covariates subsection and the statistical analysis section now refers back to this list without restating it.

**Comment 4:** The final sentence may overreach: “These findings suggest that while alcohol use is common, it may not directly impact HIV outcomes.” Evidence suggests alcohol can directly impact outcomes even with ART adherence. Please consider reframing and suggesting mediation analyses.

**Response:** We appreciate this important point. We have revised this section to avoid overstatement and instead emphasize the relationship is more complex in the modern ART era and require further mediation analyses.
